# Optical dating in a new light: A direct, non-destructive probe of trapped electrons

**DOI:** 10.1038/s41598-017-10174-8

**Published:** 2017-09-26

**Authors:** Amit Kumar Prasad, Nigel R. J. Poolton, Myungho Kook, Mayank Jain

**Affiliations:** 1 Center for Nuclear Technologies, Technical University of Denmark, DTU Risø Campus, Roskilde - 4000, Denmark; 2 Camlin Technologies Ltd, 31 Ferguson Drive, Lisburn, County Antrim, BT28 2EX United Kingdom; 3 Present Address: Schulich Faculty of Chemistry and Solid State Institute, Technion – Israel Institute of Technology, Haifa, 32000 Israel

## Abstract

Optical dating has revolutionized our understanding of Global climate change, Earth surface processes, and human evolution and dispersal over the last ~500 ka. Optical dating is based on an anti-Stokes photon emission generated by electron-hole recombination within quartz or feldspar; it relies, by default, on destructive read-out of the stored chronometric information. We present here a fundamentally new method of optical read-out of the trapped electron population in feldspar. The new signal termed as Infra-Red Photo-Luminescence (IRPL) is a Stokes emission (~1.30 eV) derived from NIR excitation (~1.40 eV) on samples previously exposed to ionizing radiation. Low temperature (7–295 K) spectroscopic and time-resolved investigations suggest that IRPL is generated from excited-to-ground state relaxation within the principal (dosimetry) trap. Since IRPL can be induced even in traps remote from recombination centers, it is likely to contain a stable (non-fading), steady-state component. While IRPL is a powerful tool to understand details of the electron-trapping center, it provides a novel, alternative approach to trapped-charge dating based on direct, non-destructive probing of chronometric information. The possibility of repeated readout of IRPL from individual traps will open opportunities for dating at sub-micron spatial resolution, thus, marking a step change in the optical dating technology.

## Introduction

Geochronology based on optical methods has played a critical role towards understanding past climates, environments, landscapes, and human evolution and dispersal in the last 0.5 Ma^1–3^. Optical dating, using Optically Stimulated Luminescence (OSL) was first proposed and demonstrated by Huntley *et al*.^4^, and it uses the light-sensitive electron traps in commonly available minerals on the Earth’s crust such as quartz and feldspars. Soon after the discovery of OSL in quartz^4^, a new optical dating signal in feldspar using Infra-Red Stimulated Luminescence (IRSL) was discovered by Hutt *et al*.^5^. The last four decades of research in OSL dating have seen several exciting innovations in the measurement and use of OSL and IRSL signals for increased precision, accuracy and age range, as well as new, challenging applications in thermochronology^6^, exposure dating^7^ and sediment transport^1,8^.

Naturally, occurring wide bandgap minerals such as quartz and feldspar contain defects (traps), which capture free electrons and holes when exposed to ambient ionising radiation in a sediment layer. Optical dating relies on the release of trapped electrons (or holes) by visible or near infra-red photons leading to an anti-Stokes photon emission generated by electron-hole recombination. The intensity of the emitted signal (OSL or IRSL) is a function of the overall population of the trapped charge within the crystal lattice, which in turn is a function of how long the crystal was exposed to ionising radiation since its burial in a sediment layer; this time duration denotes the depositional age of that sediment layer. In practice, OSL dating involves separation of quartz or feldspars from the sediment sample followed by stimulation of quartz with green (~2.3 eV) or blue photons (~2.64 eV), or feldspar with near infrared photons (~1.4 eV) to obtain the OSL and IRSL respectively^9^. From these signals the absorbed dose from ionising radiations (unit Gy = J/kg) is deduced by building a luminescence dose-response curve using a laboratory ionising radiation source. Age is calculated by dividing the absorbed dose by the dose rate; the latter is estimated through the assay of radioactivity in the sediment and cosmic ray influx.

**Figure 1:**
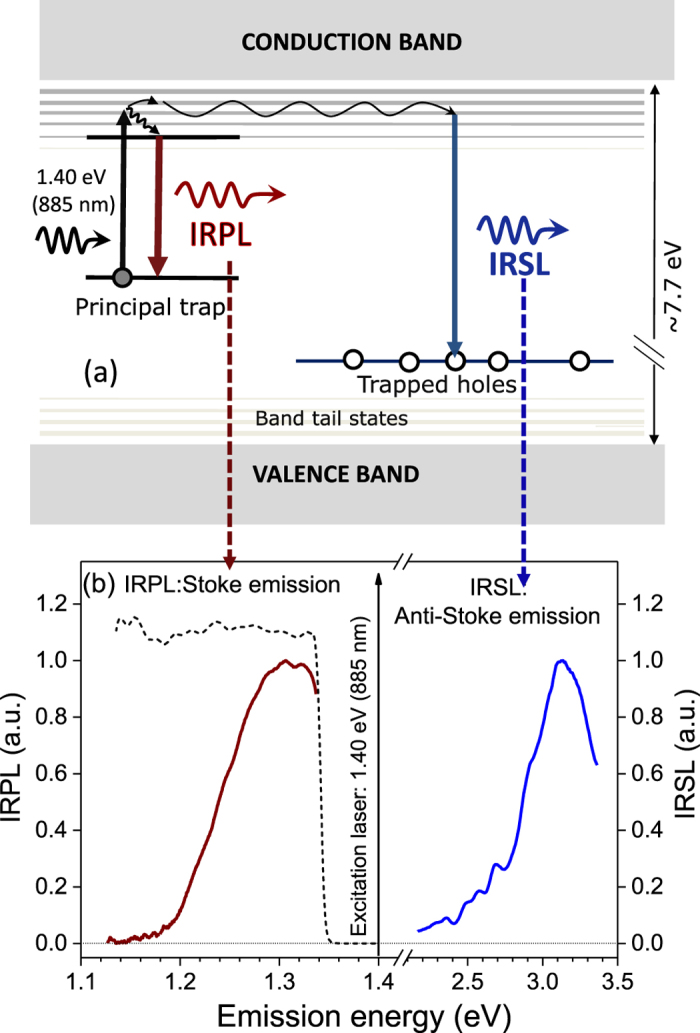
(**a**) A schematic showing the mechanisms of Infra-red Photoluminescence (IRPL) and Infra-red stimulated luminescence (IRSL) generation in feldspar. The IRSL results from recombination of de-trapped electrons with trapped holes in the crystal, whereas the IRPL is produced when the excited electron de-excites to the ground states of the same principal trap. (**b**) IRPL and IRSL emission spectra at 295 K of irradiated sedimentary K-feldspar R47 on exposure to 1.40 eV (885 nm) photons. The dotted curve represents the transmission of the long- pass interference filter used for IRPL detection.

The fundamental mechanism of the OSL or IRSL measurement technique is the eviction of trapped electrons by optical excitation, and subsequent radiative recombination of these electrons with trapped holes in the crystal. Figure 1(a) illustrates the accepted mechanism of IRSL production in a feldspar^10^. Infra-red light excites a trapped electron to a higher energy state within the principal trap (i.e. the dosimetric trap with the characteristic resonance excitation at ~1.4 eV)^5,11–22^; this is followed by electron diffusion within the band tail states and radiative recombination with a hole trapped elsewhere in the lattice^20–22^. This mechanism implies that OSL/IRSL readout by default relies on destruction of the chronometric/dosimetric information (i.e. electron and hole annihilation) stored in the crystal. In other words, OSL/IRSL is by default a destructive-readout technique where the trapped electron population decreases over the stimulation time because of their recombination with holes. This inherent destructive aspect of OSL/IRSL measurement requires that the luminescence readout be as efficient as possible (since the population of trapped electrons decreases over stimulation time), and that any irreversible (sensitivity) changes between the readout of the natural and the laboratory generated OSL/IRSL signals are corrected for. In contrast, an alternative trapped charge dating technique, electron-spin-resonance (ESR), can directly detect the trapped electrons in a non-destructive manner; however, the ESR dating method has had a restricted application in sediment dating because of the difficulty of optically zeroing the signal prior to burial^23^. Furthermore, ESR dating is not applicable to feldspars because of the lack of a readily identifiable dosimetric signal.

Feldspars are particularly attractive for geochronometry since they have an extended dating range compared to quartz. For the typical dose rate in nature (~2 Gy/ka), the trap reservoir that gives rise to IRSL in feldspar saturates at ~0.5 Ma compared to only about ~0.1 Ma in quartz. However, historically, this potential of IRSL has not been exploited because of the loss of trapped charge with time due to quantum mechanical tunnelling, a process commonly referred to as anomalous fading^24,25^; this loss of trapped electrons gives rise to age underestimation. In recent years, the problem has been tackled with some success by correcting for the fading loss^26,27^, and/or selectively sampling electron-hole populations that are less prone to fading recombination^10,18,28–32^. It is debated whether the more stable variants of IRSL, known as post IR-IRSL (pIR-IRSL)^30^, MET-IRSL^29,32^ or pulsed IRSL (PIRSL)^31,33^, exhibit fading over geological time scales or not^18,28^. Furthermore, pIR-IRSL and MET-IRSL signals do not reset as rapidly as IRSL on exposure to daylight, and suffer from undesirable effects such as thermal transfer during heating and optical stimulations at high temperatures (e.g., pIR-IRSL at 290 °C)^28,34^. Pulsed IRSL does not suffer from the problems of bleachability (signal resetting by exposure to daylight) and thermal transfer^18^; however, luminescence sensitivity is an issue here since more than 90% of the luminescence is deliberately rejected in the readout.

Given the inherent potential of feldspar dating in extending the dating age range, it is desirable that new methods be explored that do not suffer from the above problems. Models of IRSL in feldspar suggest that there is a significant relaxation of the electrons from the excited to the ground state of the principal trap, and that this process must dominate over electron-hole recombination rate via tunneling^16,17,20,21^. Similarly, work on IR radio-fluorescence (luminescence emitted during exposure to a source of ionising radiation; IR- RF) suggests that the NIR emission (~1.44 eV) arises from a radiative transition during the trapping of electrons from the conduction band into the principal trap^35^. It has also been observed that there is a delayed luminescence (phosphorescence) emission from irradiated feldspar in the range 1.26 to 1.38 eV, which has been speculated to arise from retrapping within the principal trap^13,36^. Combining these experimental observations with the IRSL model suggests that IR excitation must lead to a luminescence emission arising from radiative relaxation of electrons from the excited to the ground state of the principal trap. Such a signal for example has been shown in model systems (analogues to feldspar) such as YPO_4_: Ce, Sm where it is indeed possible to measure dose-dependent, but time-stable luminescence by monitoring radiative relaxation within the metastable Sm^2+^ (the electron trap)^37,38^. With this background, we explore for the first time the Stokes photoluminescence produced from radiative relaxation within the principal trap in feldspar previously exposed to ionising radiation. We demonstrate that this new signal is a steady state signal, i.e. it can be measured non-destructively, and it may be used for sediment dating.

## Results and Discussion

### Internal transition in the principal trap: IR Photoluminescence (IRPL)

Hutt *et al*.^5^ discovered a broad resonance at an excitation of ~1.45 eV for the principal trap in feldspar; the exact defect responsible for the principal trap is not yet conformed^39^, but it deduced to be associated with the T1 site^40^. A detailed survey of this resonance on a variety of samples has shown that the excitation spectrum of the principal trap consists of two to three overlapping peaks^12^. The temperature dependence of these peaks suggests that the electron-hole recombination is facilitated by transport through the band tail states^11,20^. The emissions resulting from IR (~1.45 eV) excitation have been reported to be in the UV (~3.76 eV), UV-Violet (~3.13 eV), green-orange (~2.18 eV) and red (~1.71 eV) bands^13,41^, with the UV-Violet being the most commonly used emission in IRSL dating. Notably, all these emissions occur in the anti-Stokes mode (emission energy > excitation energy); there exist no demonstration of possible Stokes emissions (emission energy < excitation energy) for dosimetry/dating, which is the focus of this study.

To explore the existence of dose-dependent, Stokes emission in feldspar, we used 25 different feldspar samples (see Table 1) comprising a mix of sediment feldspar extracts and museum samples. The sample and instrumentation details are given in the experimental section. Three samples were selected for detailed investigations: a sedimentary K-feldspar sample (R47) and two museum specimens (R28 and R51). The sedimentary samples were chosen bearing in mind that the main application of our study is in sediment dating.

After exposing R47 to X-rays for 2 hours (40 kV; W target; dose rate, 0.06 Gy.s^−1^), we excited it with a laser emitting at 1.40 eV (885 nm) at 295 K. Figure 1(b) shows the presence of a strong Stokes emission peaking at ~1.30 eV (955 nm). Briefly, the spectrum was measured with a CCD coupled to a spectrograph and a long-pass interference filter with a sharp cut-off at ~1.34 eV (925 nm) for blocking the scattered laser light. Also shown in Fig. 1(b) is the anti-Stokes IRSL emission spectrum at 295 K from the same sample with the associated mechanism shown in the Fig. 1(a); this spectrum is similar to those reported in earlier studies^13^ and consists of a dominant broad band in the near UV/violet peaking at ~3.1 eV.

**Figure 2:**
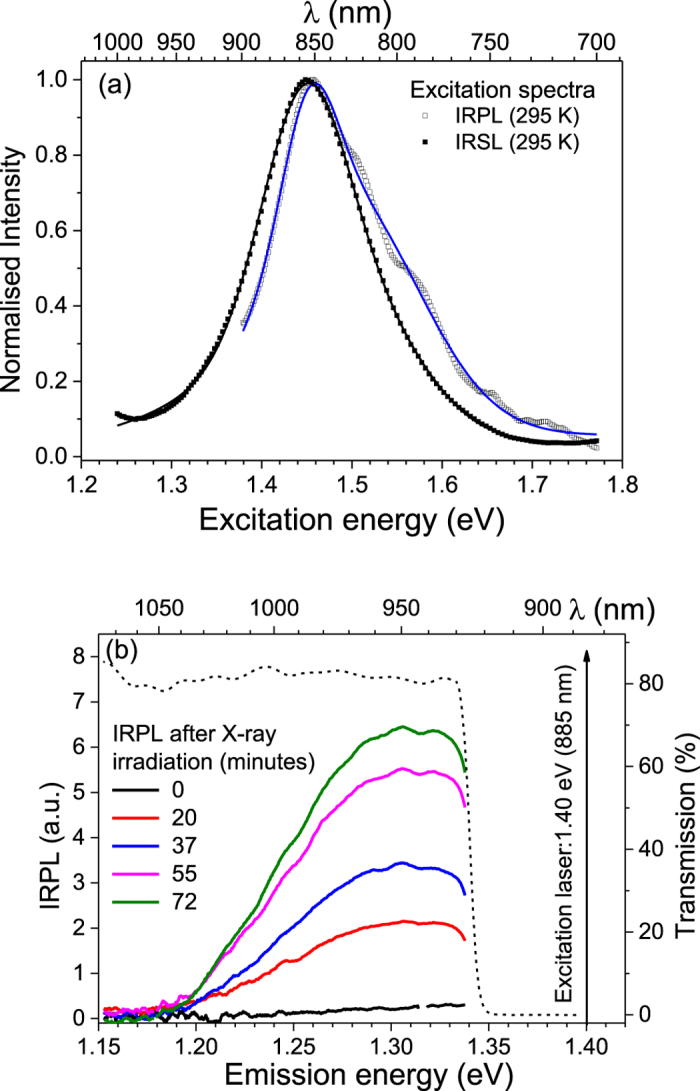
(**a**) A comparison of IRPL and IRSL excitation spectra for R47 at 295 K: the solid curves are best fits to the data, with parameters given in Table 2. (**b**) X-ray dose dependent (Dose rate: 0.06 Gy/s) IRPL spectra from sample R47 at 295 K using 1.40 eV stimulation. The dotted curve shows the transmission of the sharp-cut off long-pass interference filter, needed to block the scattered laser light.

In order to examine the characteristics of this new Stokes emission, we measured its excitation spectrum while fixing the emission window at ~1.30 eV (955 nm) (see Fig. 2(a)). For comparison, we also measured the IRSL excitation spectrum at 295 K, while fixing the emission in the UV window using a Hoya U340 filter (central wavelength: 3.65 eV or 340 nm, FWHM: 85 nm). The two excitation spectra are similar: Gaussian peak fitting gives two components (1.45 and 1.49 eV) in the Stokes emission and two components (1.45 and 1.56 eV) in the IRSL emission. There is an overlap in the dominant peak at 1.45 eV in both the spectra, strongly suggesting that the two signals are arising from the same trap.

To determine whether or not the 1.30 eV emission depends on ionising radiation dose, its intensity was measured as a function of X-ray duration. The data are shown in Fig. 2(b); there is a systematic increase in the signal intensity with prior dose, but the emission spectrum itself does not show any shift in the peak position with dose.

Based on the excitation spectrum and dose response, we interpret this new Stokes photoluminescence to arise from excitation of the trapped electron within the principal trap, followed by radiative relaxation from the excited state to the ground state (Fig. 1 - left side). The signal intensity increases with irradiation dose because of an increase in the trapped electron population. We name this Stokes emission as the **I**R **P**hoto**L**uminescence (IRPL). In the literature such an emission mechanism is commonly referred to as radio-photoluminescence^42^, however, because of the potential confusion with radioluminescence in the IR wavelength range in feldspar^39,43^, we have chosen to remove the prefix ‘radio’ from radio-photoluminescence.

In the following we further explore the physics and the dosimetric behavior of the IRPL.

### Low temperature investigations of the IRPL mechanism

In this section, we report on the low temperature characteristics of IRSL and IRPL to understand the details of the IRPL mechanism. The sample R47 was first bleached under a Solar simulator (Hönle SOL 2, commonly used for OSL dating studies) for 48 hours, and then mounted on the cold finger of a cryostat followed by *in-situ* X-irradiation at 7 K.

**Figure 3:**
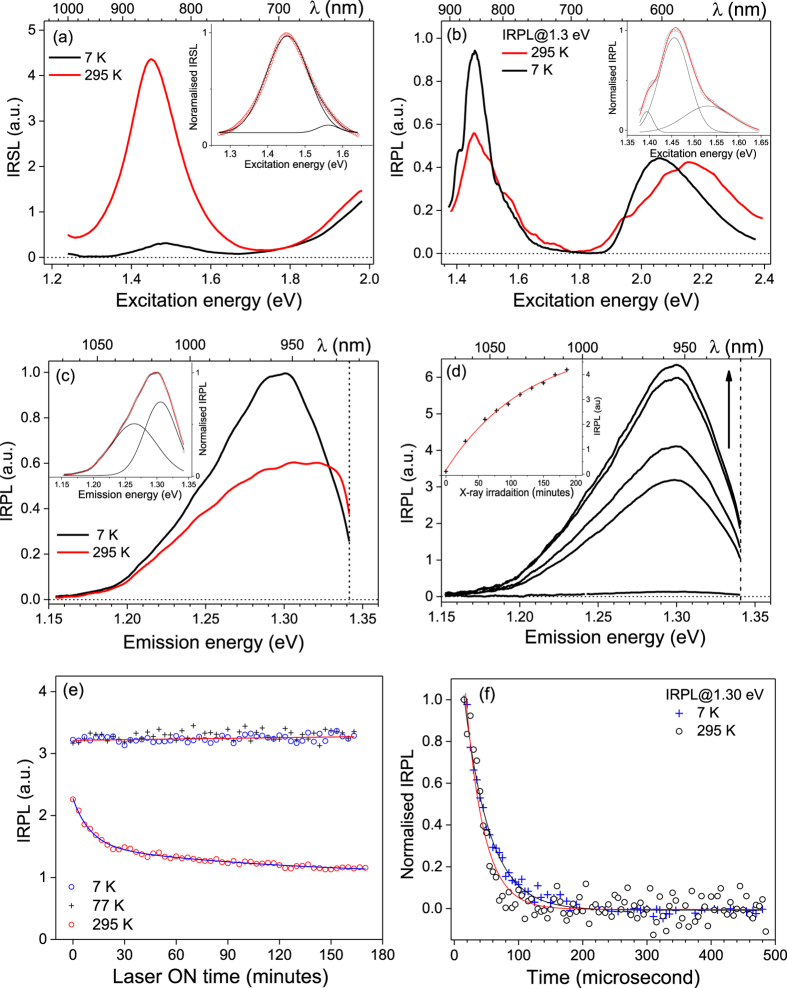
Summary of the main luminescence features for sedimentary sample R47 at 7 K and 295 K. Fitting parameters are given in Table 2. (**a**) IRSL excitation spectra after 2 hours of X-ray irradiation; the inset shows peak fitting for 295 K data. (**b**) IRPL excitation spectrum after 3 hours of X-ray irradiation, recorded for emission fixed at 1.3 eV (955 nm). The inset shows peak fitting for 7 K data in the near IR range. (**c**) IRPL emission spectrum after irradiation; the inset shows the peak fitting for 7 K data. The vertical dotted line indicates the sharp cut-off long pass filter position. (**d**) X-ray dose dependence of IRPL spectra at 7 K, under 1.40 eV (885 nm) stimulation; the upward arrow shows the intensity increase with X-ray irradiation time. The inset shows the dose response of IRPL. (**e**) Stability of IRPL under 1.40 eV, 1.5 mW/cm^2^ laser exposure. Data for 7 K and 77 K were fitted with a linear function whereas for 295 K a sum of two exponential decays was used. (**f**) The IRPL time-decay characteristics under 1.47 eV, 1.7 mW/cm^2^ laser excitation and emission at 1.3 eV (955 nm). A single exponential decay function is used for fitting.

Figure 3(a) shows the IRSL excitation spectra measured at 295 K and 7 K using the UV emission transmitted through a Hoya U340 band pass filter. At 295 K, we observe a broad peak at 867 nm (1.43 eV); Gaussian peak fitting in the excitation energy range 1.28–1.65 eV shows two overlapping bands at 1.45 eV, and 1.56 eV. The intensity at 7 K is much smaller than that at room temperature, as has been observed earlier^20^. Figure 3(b) shows the excitation spectrum of IRPL for the 1.3 eV (955 nm) emission at 7 K and 295 K; the IRPL signal shows the opposite effect compared to IRSL, i.e. the intensity at 1.45 eV increases on lowering the temperature. The IRPL excitation spectrum has two broad resonance features at ~1.455 eV and 2.1 eV. The 1.45 eV peak represents the excited state resonance of the principal trap; Gaussian fitting of the 7 K data in the range 1.37–1.65 eV shows peak component at 1.39 eV (minor), 1.455 eV (main) and 1.53 eV. The 2.1 eV peak may potentially represent the conduction band transition; the reduction in peak intensity after 2.1 eV is possibly due to an increase in the probability of electron-hole recombination (IRSL) through the conduction band relative to the probability of retrapping (IRPL production).

Figure 3(c) shows the comparison of the IRPL emission spectra measured at 7 K and 295 K using the CCD detector and excitation at 1.40 eV. The emission intensity increases by about 30% on lowering the temperature from the 295 K to 7 K, and the emission spectrum becomes narrower. Fitting of the emission at 7 K shows two overlapping bands centered at 1.26 eV and 1.31 eV.

These spectroscopic observations are consistent with our conceptual model shown in Fig. 1(a). The temperature dependence of the IRSL excitation spectra^11,20^ suggest that majority of IRSL occurs after electron diffusion within the band tail states^10,20^; the large reduction in the IRSL intensity at 7 K occurs because of freezing of electron transport in the band tail states^20^. The minor IRSL emission observed at 7 K in Fig. 3(a) could result from direct tunneling from the excited state of the principal trap to the recombination center^20^. IRPL compliments the IRSL data; the IRPL intensity is enhanced at 7 K due to reduced loss of electrons from the excited state to the band tail states, implying that a significantly greater proportion of excited state electrons can undergo relaxation to the ground state. Additionally there may be a reduced probability of thermal quenching (if any) at 7 K within the principal trap.

Figure 3(d) shows the dose response of the IRPL at 7 K. The delay between the measurements and X-ray irradiation was around 100 s, and there was no preheat before the IRPL measurement. The IRPL intensity increases with X-ray irradiation time. The inset to Fig. 3(d) shows the dose response curve (integrated peak signal vs. the X-ray irradiation time) fitted with single saturating exponential growth function. Similar to the room temperature data (Fig. 2(b)), the 7 K spectra show neither a change in shape nor a shift in peak energy with dose; the main difference between measurements made at 295 K and 7 K is the narrowing of the IRPL peak at 7 K.

We further investigated the stability of the IRPL emission when probed with the IR laser (1.40 eV) at 7 K, 77 K and 295 K. After X-ray irradiation at 7 K, the sample was excited with the 1.40 eV (885 nm) laser for about 3 hours (1.5 mW.cm^−2^ at the sample position), and the IRPL spectrum was monitored continuously during this time. Figure 3(e) shows the integrated IRPL intensity between 1.10–1.33 eV as a function of laser exposure time. There is negligible change in the IRPL intensity at 7 K and 77 K (slope: 0.0024 ± 0.0025 per hour), whereas, at 295 K there is a rapid decrease in IRPL intensity by ~40% in the first 20 minutes (fluence = 1.8 J.cm^−2^), followed by a very slow decay in the remaining time. These data suggest that the principal trap behaves like a closed system at cryogenic temperatures; the time-stable IRPL at 7 K is also consistent with there being very little IRSL emission at 7 K. Even at 77 K there is a negligible loss of electrons from the trap. However, at room temperature (Fig. 3(e)), about 40% of these electrons are lost for a cumulative IR exposure of 1.8 J.cm^−2^.The reduction in the IRPL intensity at room temperature mimics IRSL, which typically decays to a background component for ~1.8 J.cm^−2^ cumulative exposure (i.e. ~12 s exposure with a 150 mW.cm^−2^ IR source^9^). Thus, ~40% reduction in IRPL intensity at room temperature may be attributed to detrapping and subsequent loss to trapped holes to produce the IRSL signal. Based on our data it can be suggested that for R47 ~40% of the trapped electron population participates in IRSL. Since electron-hole recombination in feldspar is thought to be rate limited by the availability of proximal holes^10,17,44^, it may be argued that the remaining ~60% of the trapped electrons are sufficiently far away from any holes to undergo electron-hole recombination (IRSL production) and, therefore, not very prone to anomalous fading. While IRPL allows quantitative evaluation of the fraction of trapped electron population participating in IRSL, it also provides a non-destructive probe of dosimetric information. At cryogenic temperatures nearly all trapped electrons can be probed non-destructively because of inactive transport (i.e. no loss) through the band tail states, whereas, at room temperature, only electrons that are far enough from any holes to participate in IRSL can be probed non-destructively. From analogy with pIR-IRSL the latter population can be expected to be athermally stable (non anomalous fading electron population).

An important aspect of the IRSL process that has never been investigated earlier is the lifetime of the excited state; this parameter determines the efficiency of the IRSL signal (photons emitted per excitation photon) and is critical for the numerical models of IRSL^16,17^. The IRPL allows a way forward in determining this crucial parameter directly. Figure 3(f) shows the time decay of the IRPL measured at 7 K and 295 K using a multi-channel-scaler and a 1.47 eV (842 nm) laser modulated at 1666 Hz (pulse width = 0.4 ms, pulse energy = 37.2 µJ). The data were fitted with a single exponential decay function, which yield the IRPL lifetime of 28 ± 2 µs at 295 K and 40 ± 1 µs at 7 K. Since IRPL originates from internal transition within the principal trap, these values correspond to the lifetime of the excited state at 295 and 7 K.

The faster IRPL decay at 295 K may be due to the thermal activation of: a) non-radiative relaxation within the center commonly seen in many defects^38,45,46^, and/or b) loss of electrons from the excited state to the band tail states as evidenced in the generation of the IRSL signal at 295 K (Fig. 1(a)). Based on the IRSL excitation spectrum at 7 K (Fig. 3(a)), direct excited state tunneling to the recombination centers is expected to be a minor component^20^. The combined relative probability of these non-radiative losses at 295 K can be estimated from the lifetime data: P_nr_/(P_r_ + P_nr_) = 27%, where P_r_ + P_nr_ = 1/29 µs^−1^ (inverse lifetime at 295 K) and P_r_ ≈1/40 µs^−1^ (7 K). The 30% drop in the intensity of the initial IRPL signal in Fig. 3(e) gives further insight. If this drop in the IRPL intensity were only due to the non-radiative internal relaxation within the principal trap, then IRPL at 295 K should have been invariant with time, just like the 7 K data (see Figure 3(e) and (c)). But this is not the case; the loss in IRPL with time at 295 K, attests to the fact that loss of electrons from the excited state to the band tail states is a significant factor in determining the excited state lifetime at room temperature.

### Sample dependence of IRPL

**Figure 4:**
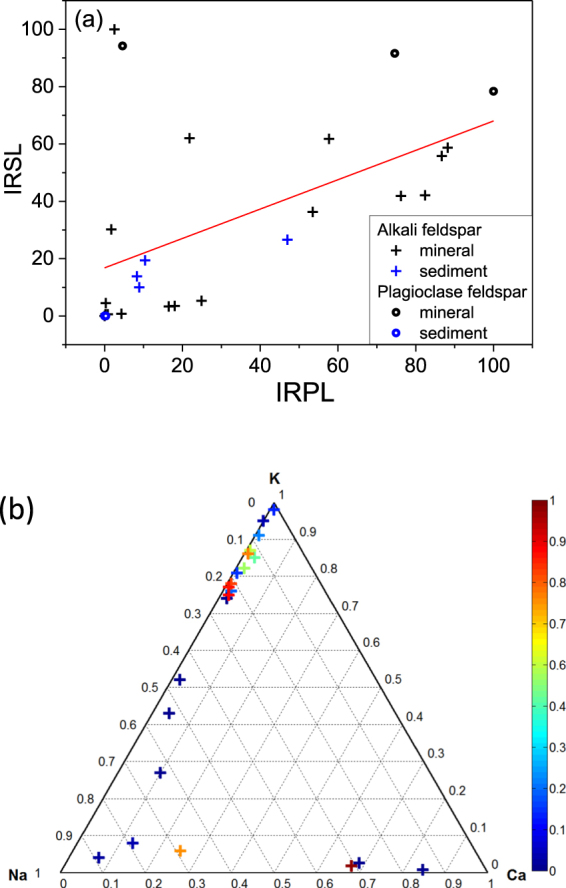
(**a**) Comparison of the normalised IRSL and IRPL for 25 different feldspar samples listed in Table 1, split between the alkali (Na/K) and plagioclase (Na/Ca) feldspar series. (**b**) Ternary diagram showing sample position and IRPL intensity as per the colour scheme in the legend.

In order to investigate the dependence of IRPL on the sample composition we measured the IRSL and IRPL for a variety of feldspar samples (See Table 1). The IRSL and IRPL signals were measured in the Risø TL/OSL reader after a dose of 700 Gy and a preheat of 250 °C for 30 s. Figure 4(a) shows the correlation between the IRSL and IRPL in samples covering both the alkali (Na/K) and plagioclase (Na/Ca) feldspar groups. The IRSL signal was integrated over a measurement time of 50 s whereas the IRPL was integrated over 220 s. There is a some correlation between the two signals (Pearson correlation coefficient, = 0.53); the differences likely attest to the variability in the concentration of hole centers and the band tail states across feldspar samples. Figure 4(b) shows the dependence of IRPL signals on feldspar composition; it appears that that IRPL sensitivity shows a slight bias towards K content.

**Table 1:** List of samples, their bulk composition as determined by XRF, and signal intensities.

Sample code	Type of Feldspar	Provenance	Composition on Unity			Normalised IRPL (%)	Normalised IRSL (%)	IRSL/IRPL ratio
			KF	NaF	CaF			
R41	M	Brazil	0.97	0.01	0.01	16.5	3.3	0.2
**R28**	**M**	**Switzerland**	**0.95**	**0.05**	**0**	**4.3**	**0.8**	**0.2**
R44	M	Spain	0.91	0.08	0.01	24.9	5.3	0.21
R45	M	Norway	0.87	0.12	0.01	57.7	61.8	1.07
R46	M	Norway	0.86	0.13	0.01	76.2	41.9	0.55
R48	M	Russia	0.83	0.16	0.02	53.5	36.3	0.68
R50	M	u/k	0.8	0.18	0.01	18	3.5	0.19
**R51**	**M**	**Norway**	**0.78**	**0.21**	**0.01**	**82.4**	**42.1**	**0.51**
R52	M	Norway	0.77	0.22	0.01	86.7	55.8	0.64
R53	M	India	0.76	0.22	0.02	21.8	62	2.84
R54	M	Norway	0.75	0.23	0.02	88.2	58.7	0.67
R55	M	u/k	0.74	0.24	0.02	0.3	4.5	15
R56	M	u/k	0.52	0.46	0.02	0.8	0.6	0.75
R57	M	u/k	0.43	0.53	0.04	0.2	0.6	3
R58	M	u/k	0.27	0.63	0.1	1.7	30.2	17.8
R59	M	India	0.04	0.89	0.07	2.5	100	40
R60	M	u/k	0.08	0.79	0.13	4.6	94.2	20.5
R61	M	Norway	0.06	0.69	0.25	74.6	91.6	1.23
R62	M	UK	0.02	0.31	0.67	100	78.4	0.78
R42	S	China	0.91	0.08	0.01	—	—	—
R43	S	China	0.91	0.08	0.01	—	—	—
**R47**	**S**	**China**	**0.85**	**0.12**	**0.03**	—	—	—
R49	S	China	0.83	0.16	0.02	—	—	—
R63	S	Iceland	0.03	0.29	0.69	—	—	—
R64	S	Iceland	0.01	0.15	0.85	—	—	—

S represents feldspars of sedimentary origin, and M represents museum single crystal samples. Here, u/k: unknown, KF: K-Feldspar, NaF: Na-Feldspar and CaF: Ca-Feldspar. The bold letters indicates that the IRPL characteristics of these samples were explored in detail (see text and supplementary infomation).

The detailed spectral, dose and time-resolved measurements presented above have concentrated on the K-rich sedimentary feldspar R47. In order to show that the characteristics are not unique to that sample, complimentary measurements are now described on two further K-rich samples (which are available as supplementary information (SI)): (i) a single crystal museum specimen (R28; dimensions 2 × 2 × 1 mm) and (ii) a second mineral specimen, R51 (gently crushed to obtain grains of 180–250 µm). We have chosen K rich samples, because of their common use in sediment dating. R28 in particular has been used previously for detailed spectroscopic measurements of the IRSL signal^11,19,20^. The data are shown in supplementary information Fig. SI-1 for R51 and SI-2 for R28, and the fitting parameters for the excitation, emission and lifetime data are summarized in Table 2. The general conclusion from these measurements is that the IRPL shows a similar behavior in our three samples (R47, R51 and R28) investigated in detail.

**Table 2:** Summary of fitting parameters for the samples R47, R51 and R28. Peak fittings shown in bold indicate the main (dominant) peak.

Experiments	Temperature	Feldspar samples		
		R47 (Sediment)	R51 (Mineral)	R28 (Mineral)
**IRSL excitation spectrum**	295 K	**1.453 ± 0.003 eV** 1.56 ± 0.01 eV	1.32 ± 0.01 eV 1.372 ± 0.004 eV **1.446 ± 0.008 eV** 1.49 ± 0.09 eV	1.358 ± 0.002 eV **1.453 ± 0.004 eV** 1.527 ± 0.008 eV
**IRPL excitation spectrum**	7 K	1.394 ± 0.006 eV **1.455 ± 0.009 eV** 1.53 ± 0.01 eV	1.399 ± 0.005 eV **1.458 ± 0.003 eV** 1.541 ± 0.003 eV	**1.463 ± 0.005 eV** 1.568 ± 0.008 eV
**IRPL emission spectrum**	7 K	1.258 ± 0.001 eV **1.305 ± 0.003 eV**	1.223 ± 0.003 eV **1.286 ± 0.001 eV**	1.232 ± 0.001 eV **1.304 ± 0.002 eV**
**IRPL lifetime**	7 K	40 ± 1 µs	47 ± 2 µs	48 ± 4 µs
**IRPL lifetime**	295 K	28 ± 2 µs	29 ± 4 µs	41 ± 7 µs

**Figure 5:**
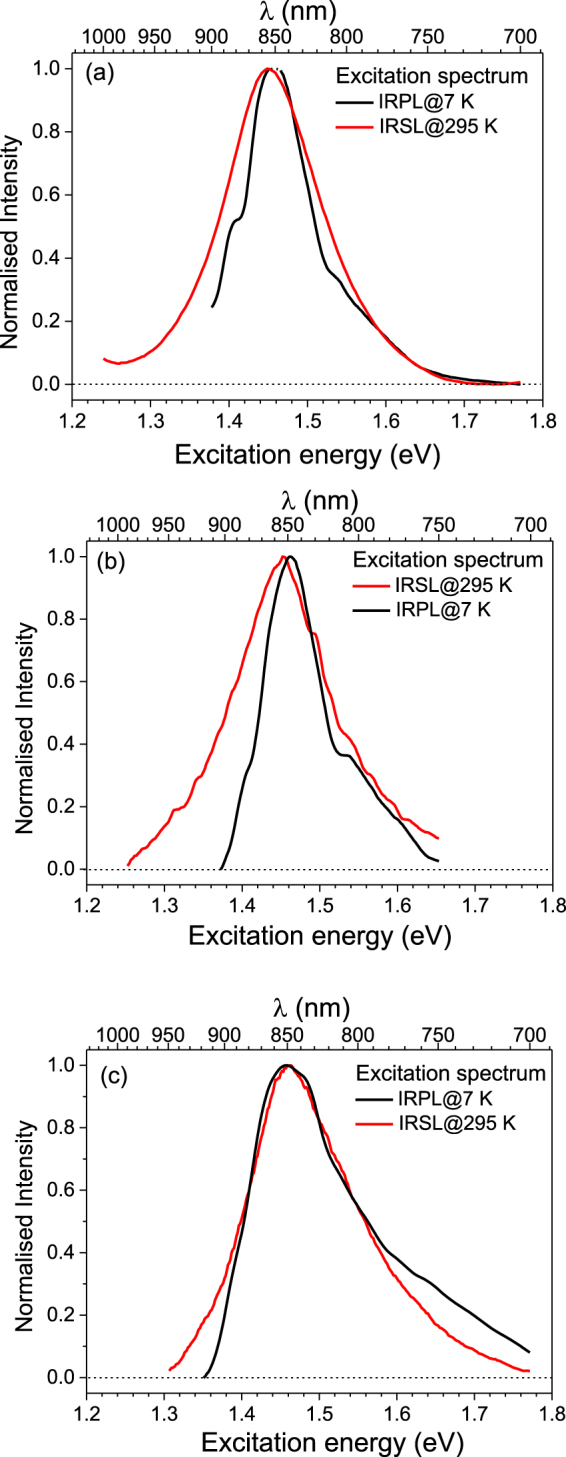
Comparison of excitation spectra of IRPL at 7 K and IRSL at 295 K for (**a**) R47, (**b**) R51 and (**c**) R28.

A comparison of the IRSL (295 K) and IRPL (7 K) excitation spectra for the three samples, R47, R51 and R28, are presented in Fig. 5(a–c) respectively. There is a good agreement between the excitation resonance for the IRPL and IRSL. Interestingly, there is a tendency for an overlap between the high energy side of the peaks, while at the lower energy side the IRPL falls off more rapidly than the IRSL. This suggests that there may be another resonance level corresponding to the second excited state at greater1.6 eV (seen more clearly in the sample R28).

### Application of IRPL in optical dating

In this section, we make preliminary investigations on the potential of IRPL for dosimetry and dating. We first test whether IRPL is amenable to single-aliquot additive dose and regenerative dose protocols that are commonly used in optical dating. We then measure the paleodose on a sample previously dated using pIR-IRSL (290 °C), and check the bleachability of the IRPL signal under SOL 2 exposure. The purpose of these measurements is to make a preliminary characterisation of IRPL for optical dating.

### Single - aliquot additive-dose-response curve

**Figure 6:**
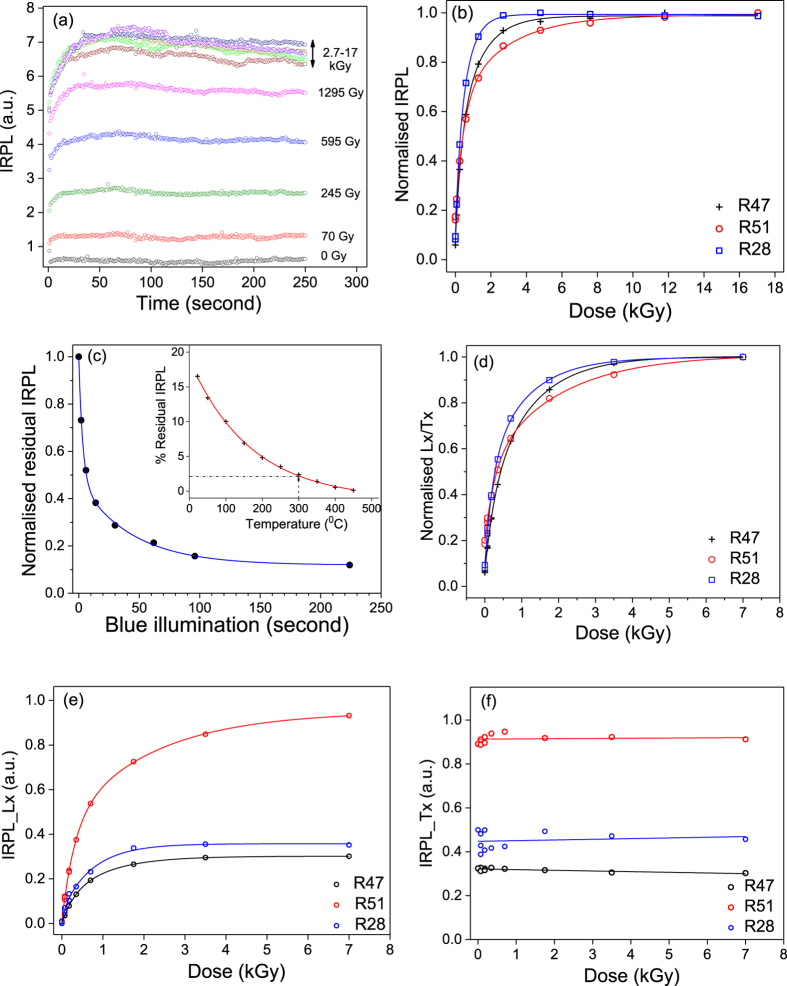
Summary and comparison of the dose and bleaching properties of IRPL in 3 representative K-feldspars; R47 and R51, and single crystal R28. (**a**) Dose dependent IRPL in R47 at 295 K as a function of the laser (1.4 eV, 885 nm) stimulation time. (**b**) Additive beta dose response of the samples; the points represent the time integral over the first 250 s of laser exposure. (**c**) The residual IRPL as a function of blue illumination time at 295 K. Inset: Residual IRPL (test dose normalised) after blue illumination for 100 s at different temperatures (using IRPL laboratory bleaching protocol Table 3). (**d**) The SAR dose response of the samples using the protocol in Table 4. Individual Lx and Tx data are shown in Fig. 6(e) and (f), respectively. (**e**) Regenerative dose IRPL (Lx) of the samples as a function of beta dose. (**f**) Test dose IRPL (Tx) of the samples as a function of prior regeneration dose.

We measured the single-aliquot additive-dose-response curves for an aliquot of sample R47. The aliquot was bleached under the SOL 2 for ~24 hours and thereafter heated at 450 °C for 100 s, to make sure that the principal trap has been emptied. It was then given beta dose (70 Gy) followed by preheat at 250 °C for 60 s and IRPL measurement at room temperature for 250 s. This cycle of beta irradiation-preheat-IRPL was repeated until a cumulative dose of 17 kGy was achieved. The time and dose dependence of the IRPL are shown in Fig. 6(a). The IRPL increases up to about 3.7 kGy of added dose, after which there is no detectable increase. The time-dependent IRPL shows an initial rise in intensity during the first 20 s for additive doses up to 1.3 kGy (because the excitation laser intensity initially rises and becomes stable during this time), after which the IRPL appears to be constant. At doses above 1.3 kGy, there is about 10% reduction in the intensity between 50–250 s of stimulation. These data suggest that the principal trap quite simply undergoes excitation-relaxation transitions upon stimulation with 1.40 eV, without any significant loss of trapped electrons: note that the depletion seen in Fig. 3(e) occurs over 20 minutes. Figure 6(b) shows the additive dose response curves for our three samples (1 aliquot each) measured in the same manner; the signal in R47 reaches a saturation level at about 2 kGy, whereas in R51 and R28 the saturation level occurs around 5 kGy.

The important conclusion of these measurements are: a) it is easily possible to measure IRPL signal without worrying about signal depletion (an apparent 10% depletion occurs only at very high doses that are not useful for dose estimation in any case), and b) that all the samples show the expected saturating exponential dose response behavior with a dynamic range that is similar to IRSL.

### Single-aliquot regenerative-dose-response curve

The Single-Aliquot Regenerative (SAR) dose protocol is currently the preferred method for quartz^47^ and feldspar dating^48^ since dose is measured using interpolation. The aim here is to find out if the IRPL signal is suitable for SAR measurements. In order to use the SAR procedure, it is important that the signal can be zeroed prior to giving a regeneration dose for building a dose response curve, or a test dose for compensating for possible sensitivity changes.

We first explored the bleaching of the IRPL using blue light. One aliquot was exposed to blue light at 450 °C for 100 s (this emptied the principal trap). It was and then given a beta dose of 700 Gy and a preheat of 250 °C for 60 s before measurement of the IRPL signal for 100 s. The aliquot was then sequentially exposed to blue light (2.64 eV, ~72 mW.cm^−2^) for 2^n^ s at room temperature followed by measurement of IRPL. The cycle of blue light exposure (n varied sequentially from 1 to 7 in consecutive cycles and IRPL) was repeated seven times until a cumulative blue light illumination of 224 s. Figure 6(c) shows the depletion of the IRPL with blue light exposure at room temperature; there appear to be two components to this depletion (the data are fitted to a double exponential function); an initial rapid depletion within a few seconds and a slower depletion in tens of seconds reaching a steady state value at $>$225 s. These components possibly represent bleachability of trapped electrons depending on the distance between the trapped electrons and their nearest hole sites. At the end of 100 s blue light exposure there is still ~15% remaining IRPL. This residual level is quite high for SAR, therefore, we investigated how the residual IRPL can be further reduced by changing the temperature for blue light stimulation.

The residual IRPL signal after 100 s of blue light exposure at various temperatures was investigated following the cycle defined in Table 3. The same aliquot as above was first exposed to blue light at 450 °C for 100 s to zero any IRPL signal followed by a beta dose of 700 Gy and a preheat of 250 °C for 60 s. Step 5 measures the IRPL response to the beta dose (without any blue light stimulation), while the Step-7 measures the remaining IRPL after blue light stimulation for 100 s at a given temperature. As shown earlier, there was no detectable depletion of IRPL in a 100 s readout, see Fig. 6(a); thus, the ratio of the IRPL in Step-7 and Step-5 (Fig. 6(c), inset) is an estimate of the fraction of remaining IRPL due to blue light exposure at a given temperature. There is nearly complete zeroing of the IRPL for illumination of 100 s at 450 °C, and >97% bleaching at 300 °C.

**Table 3:** Protocol used for the laboratory bleaching experiment

**Step-1**	Illumination with blue light (72 mW/cm^2^) at 450 °C for 100 s
**Step-2**	IRPL (885 nm laser exposure at room temperature for 250 s)
**Step-3**	Beta irradiation (700 Gy, 2000 s beta irradiation)
**Step-4**	Preheat–250 °C for 60 s
**Step-5**	IRPL (885 nm laser exposure at room temperature for 250 s)
**Step-6**	Blue light exposure at temperature (T = 25 to 450 °C) for 100 s
**Step-7**	IRPL (885 nm laser exposure at room temperature for 250 s)

Based on the above temperature dependent bleaching data shown in Fig. 6(c) we chose 300 °C as a compromise for zeroing the signal, as we do not want to heat the sample too much to avoid sensitivity changes (these parameters need to carefully optimised in the future work). The resulting SAR protocol is outlined in Table 4 and the dose response curves from the three samples are shown in Fig. 6(d). 86% of saturation signal intensity corresponds to 1.42 kGy for R28, 1.84 kGy for R47, and 2.75 kGy for R51.

**Table 4:** The IRPL SAR protocol.

**Step-1**	Preheat (250 °C for 60 s)
**Step-2**	IRPL (885 nm laser exposure at room temperature for 250 s) **IRPL, Lx, Ln**
**Step-3**	Bleaching (blue light – 300 °C for 100 s)
**Step-4**	Test dose (350 Gy, 1000 s beta irradiation)
**Step-5**	Preheat (250 °C for 60 s)
**Step-6**	IRPL (885 nm laser exposure at room temperature for 250 s) **IRPL, Tx, Tn**
**Step-7**	Bleaching (blue light–300 °C for 100 s)
**Step-8**	Regeneration dose and repeat Step 1.

There was excellent recycling ratio within 1% of unity confirming that this SAR protocol is appropriate for the IRPL. Figure 6(e, f) shows the individual responses of the regenerative dose IRPL (L_x_) and the test dose IRPL (T_x_), respectively, as a function of dose. There is almost no sensitivity change (Fig. 6(f)) in the IRPL during the SAR measurement for all the samples. We also compare the growth curves for R47 shown in supplementary information (see Fig. SI-3(b)) based on additive dose method (Fig. 6(b)) and regenerative dose method (Fig. 6(d)), to further support that there is no sensitivity change during the measurements procedure.

One important aspect of sediment dating is the ease of zeroing of the signal in daylight. Sol 2 is commonly used as a surrogate to daylight to evaluate the bleachability of a signal. We measured the solar bleaching of IRPL over 24 hours. A set of 21 aliquots of sample R47 were prepared in the dark and then each subset of 3 aliquots was exposed in the SOL 2 for 0, 6, 30, 60, 300, 600 or 1080 minutes. Subsequently the IRPL after bleaching and the IRPL response to the test dose was measured using the protocol in Table 4 (Step-1 to Step-6). The sensitivity corrected IRPL (L_x_/T_x_) is plotted as a function of the SOL 2 exposure time in the Fig. SI-3(a) in the supplementary information. The data suggest that ~80% of IRPL was depleted after 2 minutes, 93% and after 5 hours the remaining IRPL is indistinguishable from background. We also made a preliminary investigation of laboratory fading; the data are presented in Fig. SI-5 (see supplementary information) and suggest a fading rate (g %) consistent with zero.

**Figure 7:**
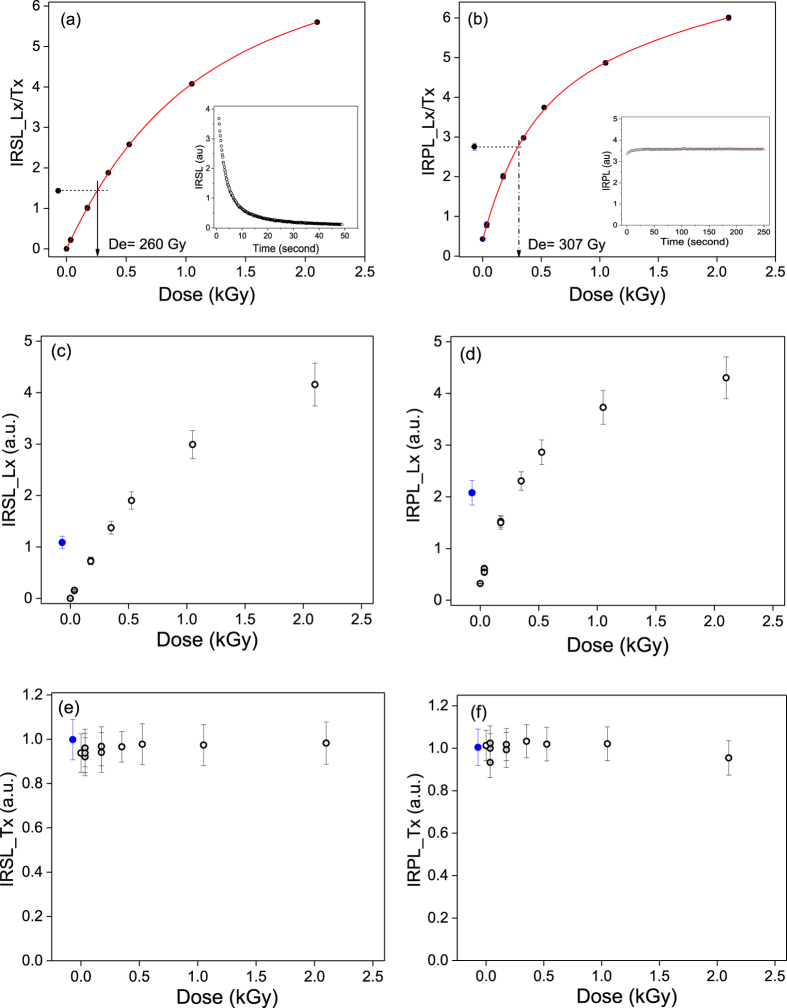
Details of dosimetry measurements for sedimentary feldspar R47. Figures (**a**) and (**b**) show the natural dose measurement via IRSL and IRPL respectively, using the dual IRSL-IRPL protocol (see Table 5); in each case, data is fitted with two saturating exponential growth functions. The Figure insets show the luminescence intensity vs. stimulation time. Figures (**c**) and (**d**) show the regenerative dose IRSL (Lx) and IRPL (Lx), respectively as a function of dose; the blue symbols represent ‘natural’ signal, (Ln). Figures (**e**) and (**f**) show the test dose IRSL (Tx) and IRPL (Tx), respectively, as a function of the corresponding regeneration dose in the SAR cycle; blue symbols represent the natural signals, Tn.

Finally, to test of the usability of IRPL for sediment dating, we measured palaeodose in fresh aliquots of the sample R47 (n = 3); these aliquots have not been previously exposed to light and have retained the trapped charge due to natural dose accumulated during deposition. As a precaution, we modified the SAR sequence to include an IR exposure (LED’s, 1.42 ± 0.03 eV, 180 mW.cm^−2^) for 50 s prior to the IRPL measurement; this additional step is to minimise any unstable component, howsoever small, in the IRPL (analogous to pIR-IRSL). The dual IRSL-IRPL protocol is outlined in Table 5. Figure 7(a) shows the dose response of IRSL (Step-2/Step-7), while the inset shows the natural IRSL signal decay. Figure 7(b) shows the dose response of IRPL (Step-3/Step-8), while the inset shows the steady state IRPL from the dose in nature. The equivalent dose using IRSL measurement was ~260 ± 6 Gy, while that using the IRPL was ~307 ± 20 Gy. The IRPL dose is consistent with the dose of ~284 ± 9 Gy measured on this sample using the pIRIR 290 °C protocol widely used to measure the stable signal (not influenced by anomalous fading) in feldspar^28,30^. Figure 7(c) and (d) show the individual responses of the regenerative IRSL and IRPL (Lx), whereas, Fig. 7(e) and (f) show the test dose response of IRSL and IRPL (Tx), respectively. The test dose intensity suggests that there is undetectable IRPL sensitivity change between the natural and the regenerative SAR cycles.

**Table 5:** The dual IRSL-IRPL SAR protocol.

**Step-1**	Preheat (250 °C for 60 s)
**Step-2**	IRSL at room temperature for 50 s **IRSL: Lx, Ln**
**Step-3**	IRPL (885 nm laser exposure at room temperature for 250 s) **IRPL, Lx, Ln**
**Step-4**	Bleaching (blue light - 300 °C for 100 s)
**Step-5**	Test dose (350 Gy,1000 s beta irradiation)
**Step-6**	Preheat (250 °C for 60 s)
**Step-7**	IRSL at room temperature for 50 s **IRSL: Tx, Tn**
**Step-8**	IRPL (885 nm laser exposure at room temperature for 250 s) **IRPL, Tx, Tn**
**Step-9**	Bleaching (blue light–300 °C for 100 s)
**Step-10**	Regeneration dose and repeat Step 1.

## Conclusion and Outlook

This study demonstrates that the principal trap in feldspar can be probed directly to read the population of trapped electrons, without depending on electron-hole recombination as in the common OSL/IRSL dating technique. The new signal reported here, Infra-red Photoluminescence (IRPL), is generated from the internal radiative relaxation within the principal trap upon excitation with the near IR light (~1.40 eV). While IRPL can be measured non-destructively at 7 K and 77 K, the time dependent IRPL data at room temperature shows an initial decaying component that participates in the IRSL, and a latter dominant component that is either stable (steady state) or decaying very slowly. Analogy with post IR-IRSL(pIRIR) techniques suggests that the initial signal should be fading prone (identical to IRSL) while the latter IRPL component should be athermally stable.

IRPL is amenable to both additive dose and regeneration dose measurement protocols for optical dating. Preliminary dosimetric investigations using time stable IRPL at room temperature (i.e. IRPL after an IRSL measurement) show that (post IR) IRPL dose is higher than IRSL and similar to that of pIRIR_290_ signal. The IRPL shows a similar dynamic dose response range and allows possibilities of preheating as IRSL; it thus does not suffer from problems faced by infra-red radiofluorescence that is also believed to result from radiative transition within the electron traps (although not experimentally demonstrated), but generated by the interaction of high energy ionising radiation with the lattice.

The IRPL signal heralds a new way of measurement of dosimetric information for optical dating; the fact that it is analogous to the ESR method, i.e., it directly probes the trapped electrons gives it several advantages over IRSL. Firstly, since IRPL is generated from electron traps irrespective of their distance to holes suggests that this signal must contain a non-fading (athermal) component. Secondly, since IRPL arises only from electron trap, it likely circumvents many issues relating to traditional OSL or IRSL methods which are sensitive to the changes in the distribution of the hole centers. Thirdly and most importantly, IRPL gives the ability to measure dosimetric signals with unprecedented precision because of the possibility of repeated readout, thus opening possibilities for 2D and  3D luminescence imaging at sub-micron spatial resolution. This possibility may give rise to exciting opportunities for luminescence dating on a sub single-grain level. Because of these expected benefits, we envision that IRPL will mark a step-change in optical dating techniques.

## Experimental Section

### Samples

A range of natural sedimentary and museum single crystal feldspar material was used, as summarized in Table 1. The feldspars themselves fall into the two common ranges; alkali (K_x_, Na_1−x_) AlSi_3_O_8_ and plagioclase Na_1−x_Ca_x_Al_1__+x_Si_3−x_O_8_. Sample compositions given are for the bulk, and take no account of potential phase segregation on the micro-scale; they were determined by XRF analysis using XRF attached with Risø TL/OSL reader. The error in individual KF, NaF, and CaF chemical content is ±0.01. The method of sediment samples preparation is reported elsewhere^49^.

## Methods

All the low temperature measurements were made using the Risø station for CryOgenic LUminescence Research (COLUR) at Center for Nuclear Technology, Technical University of Denmark, Risø﻿,﻿ Roskilde - 4000, Denmark. This facility consists of a Horiba Fluorolog-3 spectrofluorometer, upgraded to include: a temperature controlled (7–300 K) closed-loop cryostat, an *in-situ* X-ray irradiation facility (40 kV, 100 µA copper anode with 3 ms action X-ray shutter), and multiple ports for laser excitation and photo-detection, for use in a number of dual-probe type experiments. The samples are attached directly to the cryostat cold finger and measured under vacuum (2.5 × 10^−5^ mb).

Emission spectra from 910 to 1100 nm after different X-ray irradiations were measured by excitation with a 1.40 eV (885 nm), 500 mW laser and detection with Jobin-Yvon HR-320 spectrometer (300 lines/mm grating) coupled to a liquid nitrogen cooled charge-coupled devices (CCD) detector.

Excitation spectra were measured using a combination of 450 W Xe lamp and, for longer wavelengths, a 20 W tungsten halogen lamp. The excitation wavelength was selected using a double grating Czerny-Turner monochromator, while the detection was made with the same CCD system as above, but with a 925 nm long pass interference filter (optical density,OD:4) to avoid any second order and scattered light. Luminescence lifetime measurements were made using 1.47 eV (842 nm) laser diode excitation and multichannel analyser. The laser has a maximum power of 90 mW, and works in either CW mode or can be modulated with rise/fall times of < 18 ns; it was controlled via the Horiba spectrometer software.

All beta dose dependent IRPL measurements at room temperature were made with a Risø TL/OSL DA-20 reader^9,50^ with a spectrometer attachment^51^. It consists of a fibre coupled Shamrock 193i spectrograph attached to an EMCCD (iXon Ultra 888). The signal is collected through a fibre bundle consisting of 114 fibers (200 µm diameter), assembled to form a circle of 3 mm diameter towards the sample side and a rectangle towards the spectrometer side. To measure the dose dependent IRPL, the granular samples were placed in steel cups and positioned on the sample wheel of the Risø TL/OSL Reader; irradiation of the samples was achieved with Sr^90^/Y^90^ beta source (dose rate 0.35 Gy/s). The IRPL spectra were recorded by excitation with 885 nm laser and detection from 920 nm to 1050 nm. A band pass interference filter at 875 nm (FWHM: 25 nm) was set at the excitation side and a long pass interference filter with 925 nm (OD:4) was set at the detector side. All spectra shown have been corrected for instrumental response, except for the transmission of any sharp-cut long-wavelength-pass interference filters that may have been used. These filters typically cut on (10–90%) over 5 nm, and are spectrally neutral in the windows used for these experiments, but do exhibit minor oscillations in transmission due to interference fringes. The typical transmission of these filters has been included with the spectra of Figs 1(b) and 2(b) and Fig. SI-6 (supplementary information). The transmittance of the filter was recorded with Shimadzu spectrophotometer (UV-2600/2700). The IRPL spectrum in absolute counts is presented in supplementary information Fig. SI-4.

X-irradiation was achieved using a Moxtek unit with Cu anode operated at 40 kV and 100 μA; a 100 µm Aluminium foil delivering a uniform dose rate of 0.06 Gy/s on the sample of grain size of 90–180 µm. The measurement protocols related to characterising the dosimetric behaviour of the samples are summarised in Table 3 to Table 5.

## Electronic supplementary material

Supplementary information
