# Supplementary material for: Optical dating in a new light: A direct, non-destructive probe of trapped electrons
Source: Sci Rep. 2017 Sep 26;7:12097. doi: 10.1038/s41598-017-10174-8 (PMC5615069; doi:10.1038/s41598-017-10174-8)
Supplement: Supplementary file 1 — Supplementary information [file 41598_2017_10174_MOESM1_ESM.pdf]

Optical dating in a new light:
A direct, non-destructive probe of trapped electrons

Amit Kumar Prasad[1]*, Nigel R. J. Poolton[1, 2], Myungho Kook[1], Mayank Jain[1]

[1]Center for Nuclear Technologies, Technical University of Denmark

DTU Risø Campus, Roskilde-4000, Denmark

[2]Camlin Technologies Ltd, 31 Ferguson Drive, Lisburn, County Antrim, BT28 2EX

United Kingdom

(* Corresponding author: amitphotonics@gmail.com)

Supplementary information (SI)

Figure SI-1: Page number: 2-3

Figure SI-2: Page number: 4-5

Results (Figure SI-1 and SI-2): Page number: 6

Figure SI-3: Page number: 7

Figure SI-4: Page number: 8

Figure SI-5: Page number: 9

Figure SI-6: Page number: 10

Figure SI-1

Summary of the main luminescence features for mineral K-feldspar, R51 at 7 K and 295 K. Fitting parameters are given in Table 2.

(a) IRSL excitation spectra. Inset: peak fitting of the spectrum at 295 K.

(b) IRPL excitation spectrum after 3 hours of X-ray irradiation, recorded at 7 K for emission fixed at 1.30 eV (955 nm). Peak fitting is in the near IR range only.

(c) X-ray dose dependence of the IRPL spectra at 7 K, under 1.40 eV (885 nm) stimulation; the arrow indicates the intensity evolution with irradiation time. The inset shows peak fitting of the spectrum.

(d) X-ray irradiation time (dose) dependent IRPL, fitted with a single saturating exponential.

(e) Stability of IRPL at 7 K under 1.40 eV (885 nm), 1.5 mW/cm^2 laser exposure. The data was fitted with a linear function.

(f) The IRPL time-decay characteristics under 1.47 eV, 1.7 mW/cm^2 laser excitation and emission at 1.30 eV (955 nm). The data is fitted with single exponential decay function.

Figure SI-1

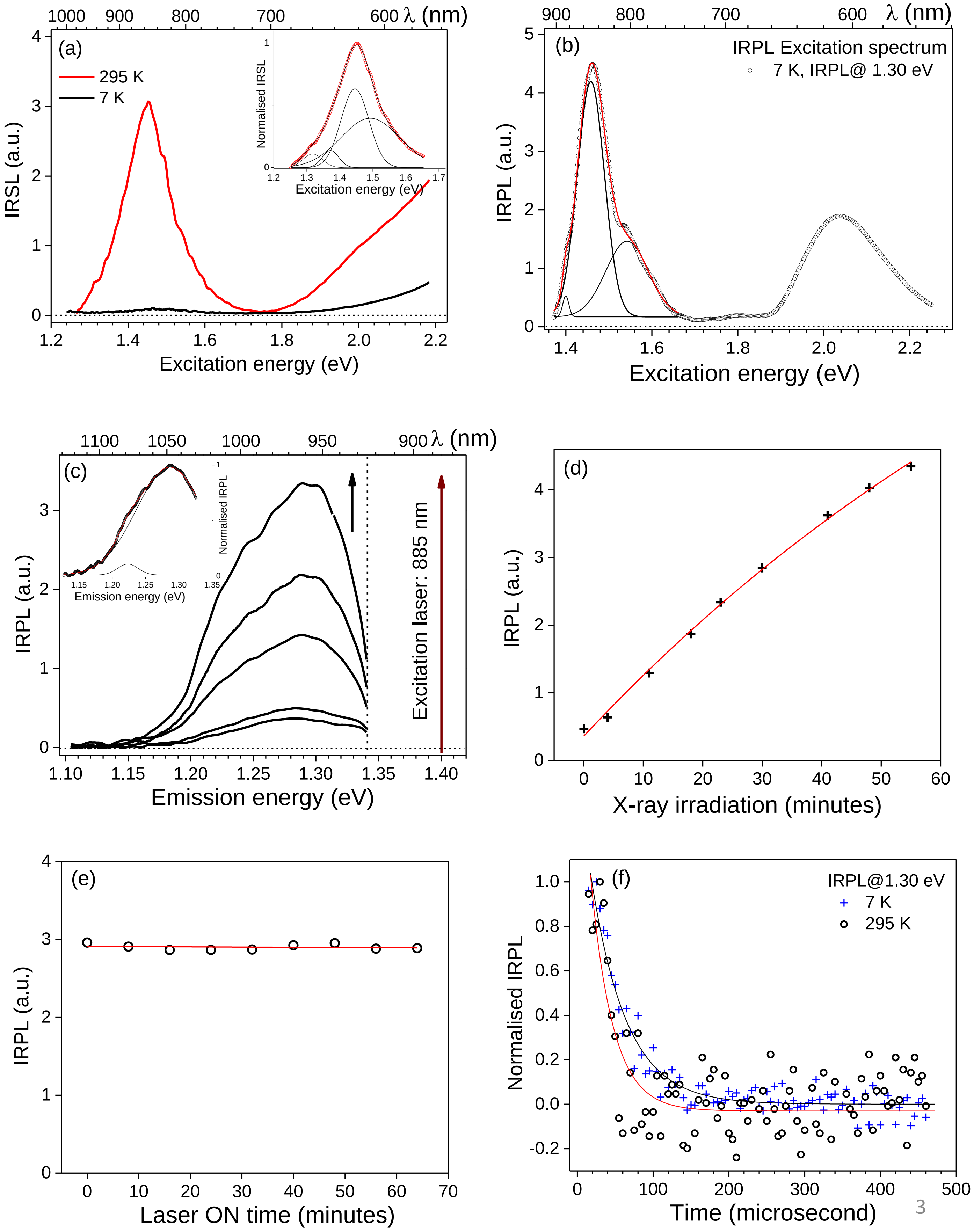
(a)
295 K
7 K
IRSL (a.u.)
Excitation energy (eV)
λ (nm)
Normalised IRSL
(b)
IRPL Excitation spectrum
7 K, IRPL@ 1.30 eV
IRPL (a.u.)
(c)
Normalised IRPL
Emission energy (eV)
Excitation laser: 885 nm
(d)
X-ray irradiation (minutes)
(e)
Laser ON time (minutes)
(f)
IRPL@1.30 eV
Normalised IRPL
Time (microsecond)

Figure SI-2

Summary of the main luminescence features for museum single crystal K-feldspar specimen R28 at 7 K and 295 K. Fitting parameters are given in Table 2.

(a) IRSL excitation spectra. Inset: peak fitting at 295 K.

(b) IRPL excitation spectrum at 7 K after 3 hours of X-ray irradiation, recorded for emission fixed at 1.30 eV (955 nm). The peak fitting is for the near IR range only.

(c) X-ray dose dependence on IRPL spectra, measured at 7 K, under 1.4 eV (885 nm) stimulation; the upward arrow shows the intensity increases as X-ray irradiation time, and curve fitting given in the inset.

(d) X-ray irradiation time (dose) dependent IRPL, fitted with a single saturating exponential.

(e) Stability of IRPL at 7 K under 1.40 eV (885 nm), 1.5 mW/cm^2 laser exposure. The data was fitted with a linear function.

(f) The IRPL time-decay characteristics under 1.47 eV, 1.7 mW/cm^2 laser excitation and emission at 1.30 eV (955 nm). The data is fitted with single exponential decay function.

Figure SI-2

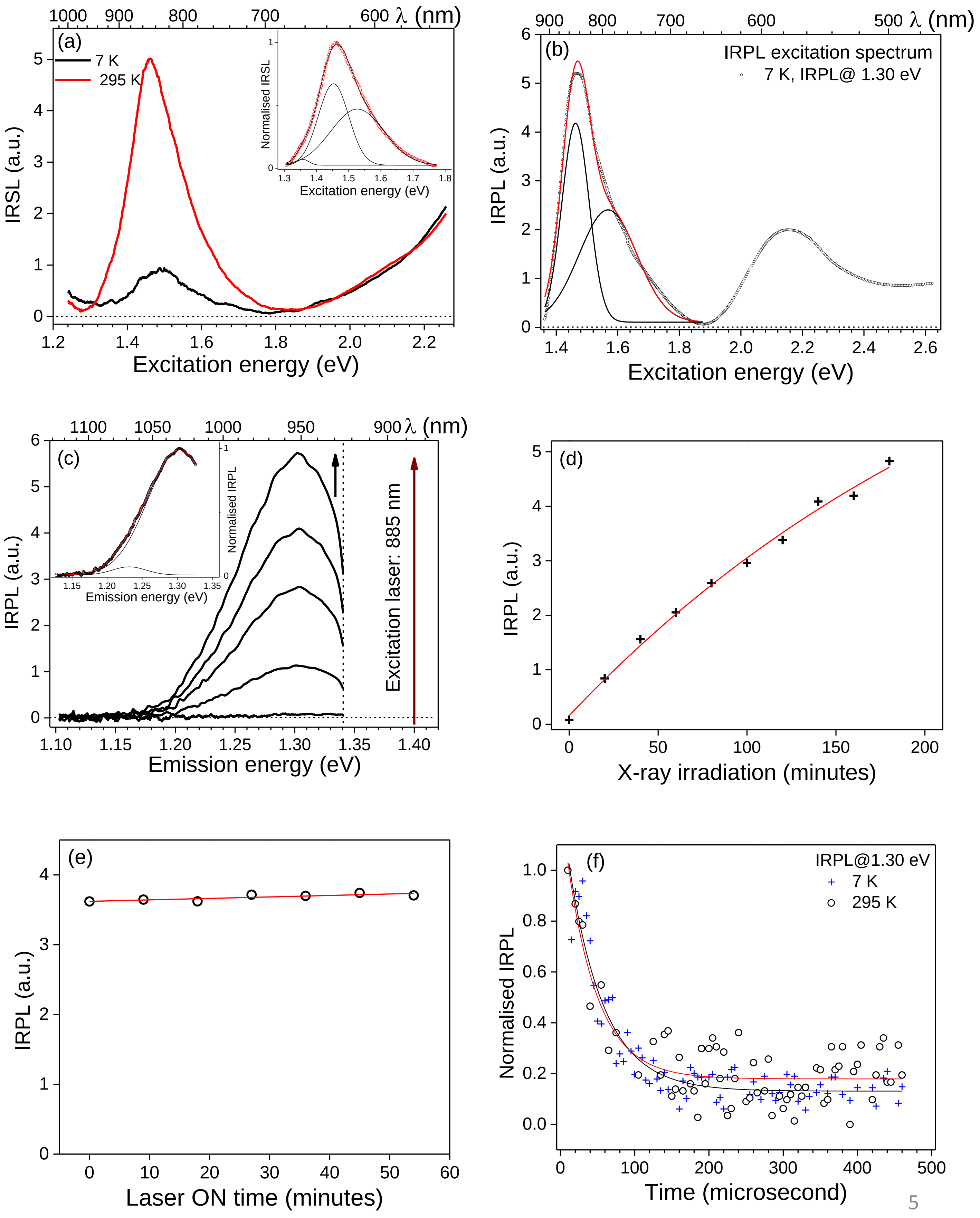

(a)
1000 900 800 700 600 λ (nm)
7 K
295 K
IRSL (a.u.)
Excitation energy (eV)
Normalised IRSL
Excitation energy (eV)
(b)
900 800 700 600 500 λ (nm)
IRPL excitation spectrum
7 K, IRPL@ 1.30 eV
IRPL (a.u.)
Excitation energy (eV)
(c)
1100 1050 1000 950 900 λ (nm)
Normalised IRPL
Emission energy (eV)
Excitation laser: 885 nm
IRPL (a.u.)
Emission energy (eV)
(d)
IRPL (a.u.)
X-ray irradiation (minutes)
(e)
IRPL (a.u.)
Laser ON time (minutes)
(f)
IRPL@1.30 eV
7 K
295 K
Normalised IRPL
Time (microsecond)

Results (Figure SI-1 and SI-2):

Figure SI-1 (a) and Figure SI-2 (a) show the IRSL excitation spectrum of R51 and R28 at 7 K and 295 K. The 295 K spectrum for both sample shows a resonance feature in the 1.25-1.65 eV for R51 and 1.3-1.78 eV for R28. The inset of these figures shows the peak fitting of IRSL excitation spectrum at 295 K. The 7 K, IRSL excitation spectrum shows a significantly reduced intensity.

Figure SI-1 (b) and Figure SI-2 (b) show the excitation spectra of IRPL emission for emission at 1.30 eV (955 nm) at 7 K for R51 and R28 respectively. The IRPL excitation spectrum for R51 shows two feature in the excitation energy range 1.35-1.65 eV, 1.9-2.2 eV, whereas, the IRPL excitation spectrum for R28 shows slightly different peak features at 1.35-1.78 eV, 2-2.4 eV. The Low energy peak, 1.35-1.65 eV for R51 and 1.35-1.78 eV for R28, fitting of the IRPL excitation spectrum is presented.

Figure SI-1 (c) and Figure SI-2 (c) shows the X-ray dose dependence of the IRPL emission spectra in the range of 1.1-1.34 eV at 7 K in R51 and R28, respectively. The inset of these figures shows the peak fitting of the IRPL emission spectrum. The integrals of these signals are plotted as a function of dose (X-ray irradiation time) in Figure SI-1 (d) and Figure SI-2 (d) respectively; the data are fitted with a single saturating exponential function.

Figure SI-1 (e) and Figure SI-2 (e) represent the IRPL emission stability on the exposure of 1.40 eV (885 nm) laser at 7 K over 60 minutes for R51 and R28 respectively, confirming its non-destructive readout characteristics.

Figure SI-1 (f) and Figure SI-2 (f) shows the temperature dependent lifetime of IRPL emission for R51 and R28 respectively. The data are fitted to single exponential functions and the derived lifetimes are summarized in Table 2.

The general conclusion from these measurements is that the IRPL shows a similar behavior in the three samples (R47, R51 and R28) (see text).

Figure SI-3

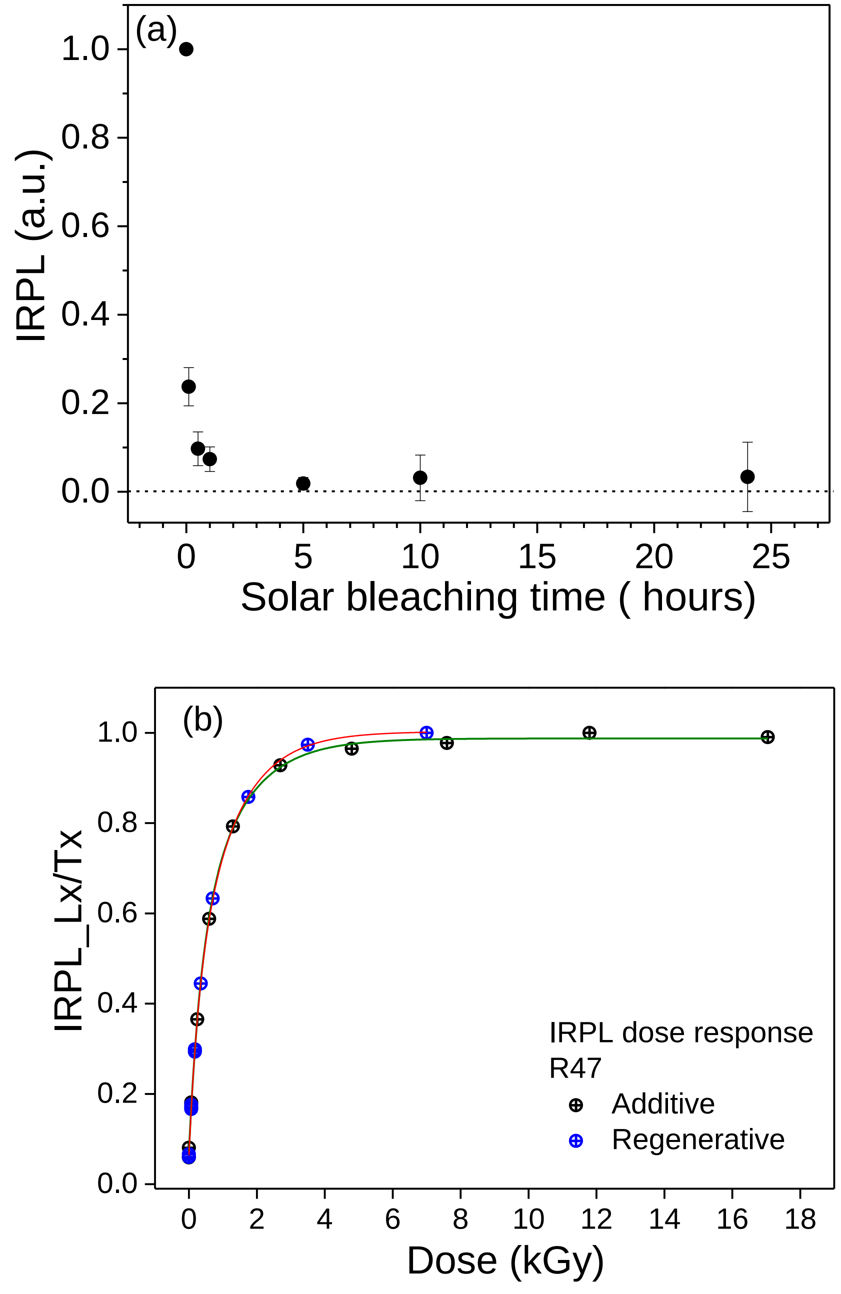

Figure SI-3.(a) Shows the bleaching characteristics of IRPL using SOL 2 (b) Comparison of the growth curves for R47 based on additive dose method (Figure 6(b)) and regenerative dose method (Figure 6(d)), to further support that there is no sensitivity change during the measurements procedure (see text).

Figure SI-4

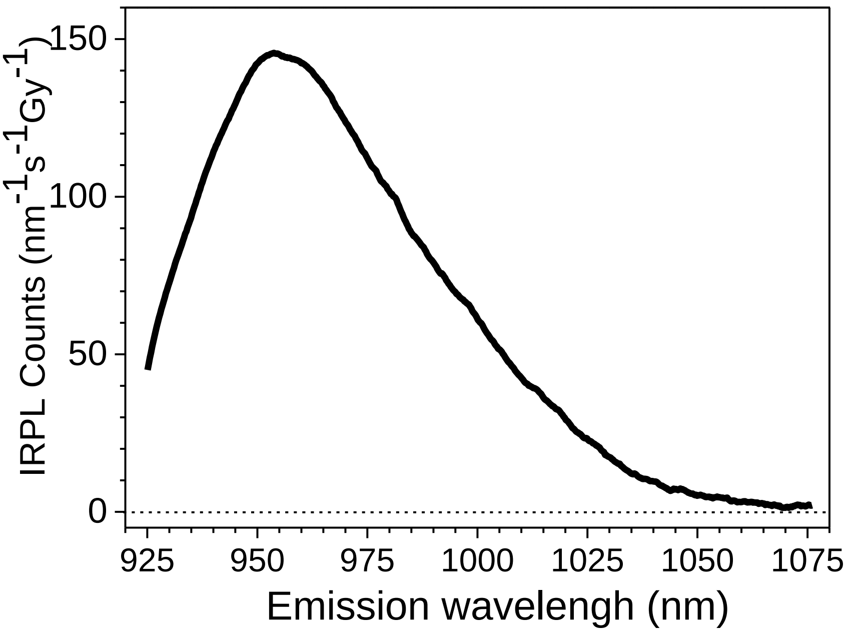

Figure SI-4. IRPL emission spectrum for R47 at 7 K plotted as number of detected photons.

Figure SI-4 shows the absolute yield of IRPL photons per unit dose per unit wavelength and time in the COLUR at 7 K for R47. Since this is a steady state signal, it is possible to accumulate it until an acceptable signal to noise ratio is achieved.

Figure SI-5

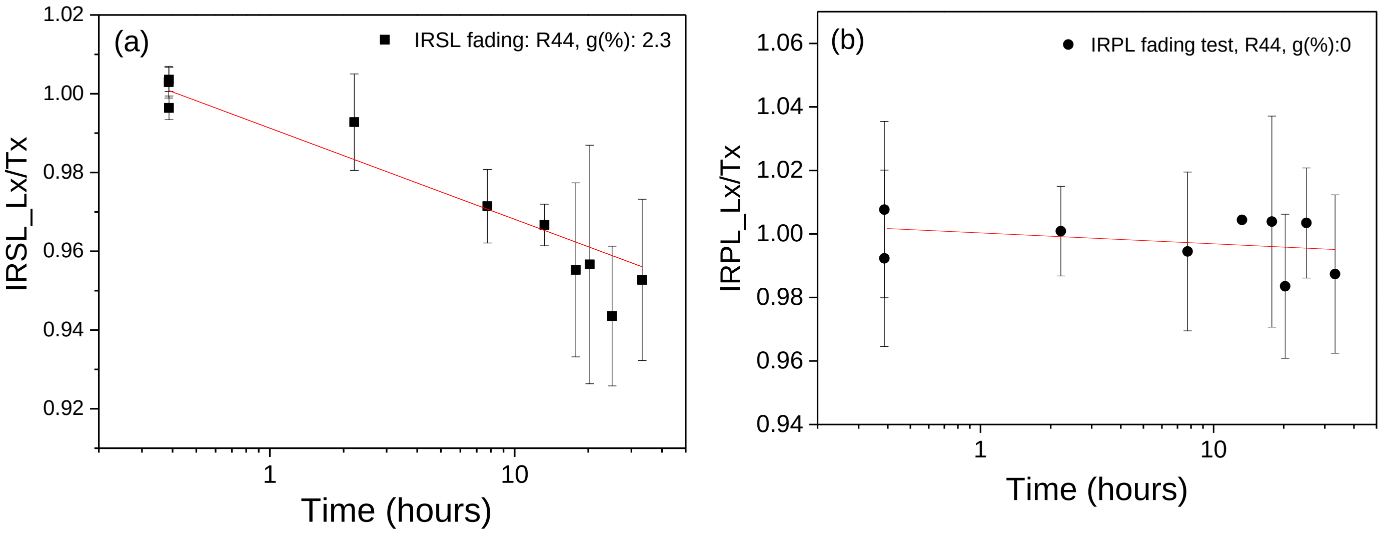

Figure SI-5. Fading experiments in R44 samples as a function of delay since irradiation (a) For IRSL and (b) for IRPL. The summary of fading rate (g %) is given in Table SI-1.

Table SI-1: Comparison of fading results (g value) using IRSL, post IR-IRSL and IRPL

Sample (3 aliquots)	g%, IR50 (preheat 320 ^{o}C for 60 s)	g%, post IR-IRSL 290 (preheat 320 ^{o}C for 60 s)	g%, IRPL at 25 ^{o}C (preheat 250 ^{o}C for 60 s)
R44	2.8 ± 0.4	0.6 ± 0.2	0 .3± 0.3
R52	1.6 ± 0.1	-0.1± 0.3	-1.0 ± 0.0

Figure SI-5 suggests that the fading rate of IRPL is consistent with zero, and it is lower than both IRSL and pIRIR290, despite the fact that a much higher preheat is used for the latter. These results are presented below in the Table SI-1.

Figure SI-6

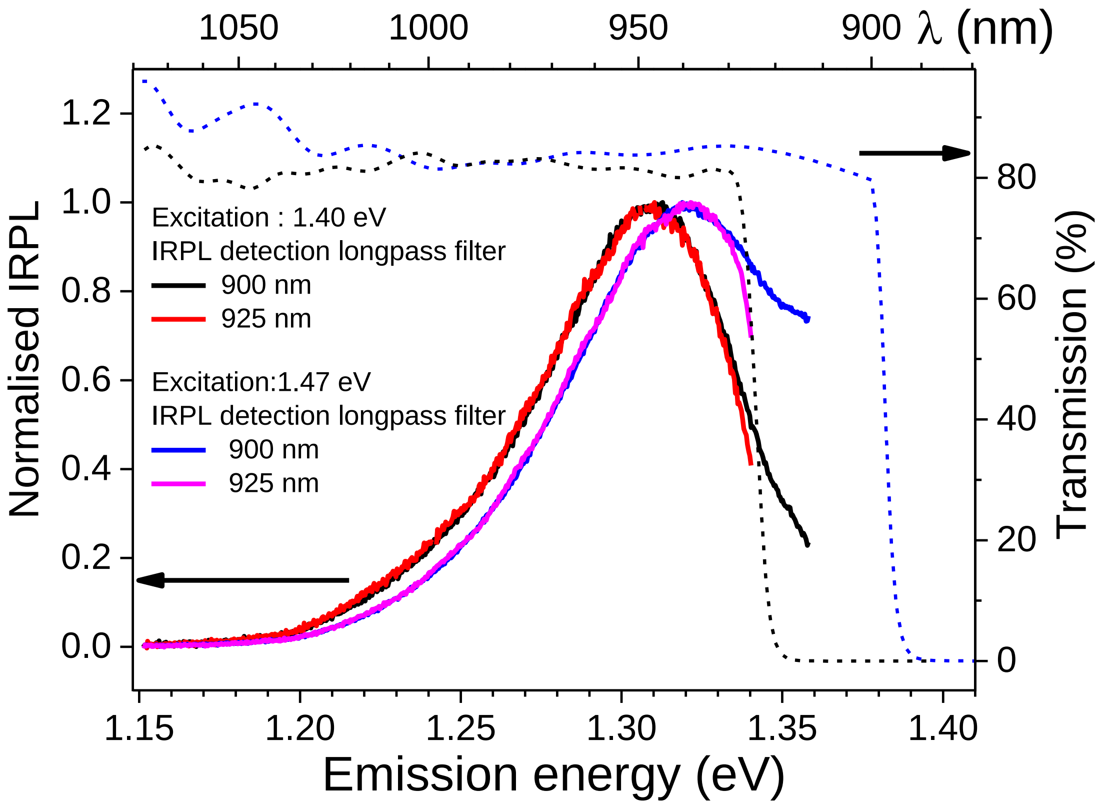

Figure SI-6. IRPL emission measured with two-detection filters (900 nm or 925 nm) and two laser excitations (1.40 eV or 1.47 eV) at 7 K for R51. Filters transmission are plotted as dashed curves.

Figure SI-6 shows that the IRPL emission peak is robust; it does not depend upon the cut-off filter used for the IRPL measurement. Furthermore, the results from two different laser excitations (1.40 or 1.47 eV) show an emission peak shift of ~ 0.1 eV for a change in excitation energy of 0.7 eV. These data confirm that the IRPL peak at ~1.30 eV is not due to Raman scattering; the slight blue shift seen here is perhaps due to the effect of the band tail states [1,2].

References:

1. Prasad, A.K. *et al.* Probing luminescence centers in Na rich feldspar. *Radiation Measurements*, **90,** 292–297 (2016).

2. Prasad, A. K. Understanding defect related luminescence processes in wide bandgap materials using low temperature multi-spectroscopic techniques. *Ph.D. thesis, DTU Nutech* (2017).
